# Africanizing scientific knowledge: the Multilateral Initiative on Malaria as a model?

**DOI:** 10.1186/1475-2875-9-S3-S7

**Published:** 2010-12-13

**Authors:** Francine Ntoumi, Gunilla Priebe

**Affiliations:** 1 Multilateral Initiative on Malaria, (AMANET Secretariat), P.O. 33207, Dar-Es-Salaam, Tanzania; 2 Institute for Tropical Medicine/University of Tübingen, Wilhemstrasse 27, 72074, Tübingen, Germany; 3 Congolese Foundation for Medical Research/University Marien Ngouabi, BP 2672, Rep of Congo; 4 Department of Philosophy, Linguistics and Theory of Science, University of Gothenburg, Sweden; 5 Department of Public Health Sciences, Karolinska Institutet, 171 77 Solna, Sweden

## Abstract

In November 2009, the fifth Pan African Malaria conference was held in Nairobi. Thirteen years after the founding initiative in Dakar, the first African Secretariat based in Africa (TANZANIA) organized this major event for the malaria community. Looking back, it has been a long way: changes in the research landscape, new funding opportunities came out and establishment of new partnerships between Europe, America and Africa. Goals identified in 1997 have not all been achieved because the critical mass of scientists has not been reached yet.  However a new generation of African scientists have emerged through MIM/TDR funding and advocacy for more support remains on the agenda. Could it be rightly stated today that the MIM concept reflects the africanization of malaria research?

## Background

Since its launch in 1997, the Multilateral Initiative on Malaria (MIM) has promoted research on malaria. Specifically, it has focused on strengthening research capacity in sub-Saharan Africa (SSA), supporting regional and international cooperation and communication, as well as the translation of research findings into policy and guidelines. The reason for targeting research capacity is that African scientists working in malaria endemic regions in many ways were marginalized, even though their contribution is critical due to their comprehensive understanding of malaria. A strong local anchorage and commitment to science is required for countries to define and address their own health research priorities. MIM asserts it is the only way towards sustainability in scientific activity.

With this focus on the relation between specific localities (malaria endemic regions) and knowledge production, MIM becomes linked to epistemological discussions within Philosophy and Theory of Science. How humans acquire knowledge has been analysed at length within these disciplines, and for example Standpoint theory analyses how the economic and political ordering of (world) society set limits to what people can “understand about themselves and the world around them” [1]. It is argued that objective membership (i.e. personal experience) in marginalized groups is a prerequisite for the comprehensive understanding of living conditions of this group.

A concept that is connected to this perspective, but specifically has been used to frame discussions about knowledge production in and about Africa and its people is “africanization”. This concept has also been related to language issues, political programs, cultural expressions, and the nationalization of natural resources, and has then been defined as the removal of colonial influence from the phenomena in question in order to give it an African character. When applied to scientific knowledge production, africanization highlights the mutlifacetted relationship between Africa and other parts of the world as well as the themes discussed within Standpoint theory.

The concept thus entails two integrated themes: (1) the meaning of locality to researchers’ ability to represent a study object correctly and with relevance, and (2) the impact of continuing colonial logics on scientific knowledge production (both in terms of epistemology and organization of research) [2].

However, the africanization concept has mainly been used in analyses of the humanities and social sciences [3][4], while health sciences (the main focus’ of MIM) still in many ways are regarded as unaffected by place or social orders. Yet, as this article shows, the concept of africanization captures the essence of MIM’s work and the issues that MIM are trying to come to terms with. It, therefore, contributes both to the understanding of the present situation and gives directions for future actions.

## Medical research in Africa in twentieth century

Medical research during the colonial era was not only under foreign influence. Rather, it was fully owned by (in relation to Africa) foreign actors: initiative for scientific research came from foreign sources, research was motivated by military strategies, and its products (e.g. quinine) were of great importance for the colonization of Africa [5]. In contemporary Africa, regional and country-specific supports to health research differ. Yet, most of the research and development (R&D) activities that has taken place after independence show that not much has changed since the colonial era. Initially, several African nation states gave priority to research: the institution that previously had served the colonial powers would now serve the African populations. These efforts were in some cases successful in the way that high quality research environments were established, but benefits in the form of development impact was not as great as expected. This together with the SAP focus on economic growth decreased state commitment to research considerably during the 1980s. Though, with the current emphasis on analytical thinking and evidence-based decisions investment into these sectors has gained renewed attention. In addition, it is obvious that the health situation remains a major issue for national authorities in SSA: more than 90 % of the world’s burden of preventable mortality occurs in poor income countries. Higher education and research capacity is today identified as fundamentals for breaking away from ill-health, for the ability to develop and implement locally based solutions to societal problems, yet also for the effective representation in international organizations. The building and strengthening of national research environments is consequently part of African states’ efforts to simultaneously enhance independence and development [6,7].

Despite of this and the fact that the burden of malaria has social and economic impact on development, health issues still do not receive enough attention in many SSA countries. In the recent past, the massive implementation of arteminsinin combination therapy (ACT) to overcome the spread of strains resistant to monotherapy drugs in the treatment of uncomplicated malaria and the succesful campaigns of indoor spraying led to the reduction or elimination of malaria in some countries [8]; however many SSA countries do not share the same success stories. Despite the real progress in funding health and specifically malaria R&D since 1970’s, the contribution of SSA countries to the world scientific publications remains non significant (approximately 1.2 %, data from 1997). Malaria R&D investment represents approximately 0.3 % of total health-related R&D investment.

## MIM mission and objectives

Since its launch, MIM has tried to handle this complex situation: it has generally advocated for more resources to malaria research, yet primarily for cooperation between researchers, and between researchers and policy makers. In addition, MIM has specifically worked to strengthen and sustain the capability of malaria endemic countries to carry out required research to develop or improve tools for malaria control. The arguments for capacity strenthening resembles those put forward in the discussions about the africanization of the social sciences. At MIM’s first conference in Dakar (1997) it became clear that many of the malaria topics of crucial importance to people living in malaria endemic regions, historically had been neglected by colonially driven research. Research topics relevant to militaries and tourists (i.e. primarily male adults temporarily visiting endemic areas) had been prioritized with consequences for the development of concepts, disciplines as well as technology [9]. Critical voices put forward that on top of this distant Western researchers that quickly “drop in to skim off results from local trials” [10] do not have such comprehensive understanding of how malaria plays out in its actual setting that is necessary for the identification of the most urgent or relevant research questions. Even though the bulk of scientific knowledge on malaria is quite extensive, it can, as in the case of the social sciences, therefore from an African point of view, be described as reductive and superficial [11][12][13]. Its base in European (laboratory-)experience of malaria rather than the every day reality of people living in malaria endemic areas, has not only had organizational but also epistemological consequences. *How* science knows malaria has had consequences for *what* it knows about malaria.

As MIM/TDR focus on funding African based research that has a clear relevance to endemic areas, it can similarly be said to re-africanize the scientific knowledge production on malaria. Many African research leaders have emerged thanks to the basic support of MIM/TDR grants. A large proportion of publications from SSA countries are output of such grants: more than 100 publications have derived from MIM funded projects in peer-reviewed journals [14]. This has contributed to highlight the new generation of African scientists, filling the gaps created by colonial educational policies. The research areas funded by MIM/TDR are diverse and may be classified in the following: pathogenesis and immunology of malaria; malaria vector control (mosquito entomology); epidemiology; insecticide resistance; chemotherapy of malaria; genetics of anti-malarial drug resistance; novel malaria control tools from traditionally used natural products, as well as research to facilitate malaria control interventions including introduction and evaluation of new strategies and policies. This exemplifies what knowledge production means when “medical narratives” are formulated from an “African point of view”. By focusing on previously neglected issues, MIM/TDR has been a tool for adding data on acquired immunity, malaria in pregnancy, indigenous knowledge on plants as well as profound knowledge about the societies where products are going to be implemented. Moreover, this is indicated in the fact that, as a requirement, MIM/TDR funded projects should be approved or at least show the relation to control programmes and the projects should be relevant for national authorities.

However, as many SSA countries still lack in research capacity and in some cases commitment from local authorities, research capacity strengthening and international cooperation is needed in parallel with the efforts to saturate research with African experiences of malaria. As was mentioned in the above africanization does not mean a rejection of exchange, as long as this exchange empowers all participants and is based on equality and mutuality. In order to meet research capacity challenges in malaria endemic areas, cooperation between researchers from different parts of Africa and other areas of the world has been encouraged. Equally important has cooperation between researchers and decision- and policy-makers, and these together with health care personnel and people experiencing malaria, been. Yet, cooperation alone (or more research alone, for that matter) has not been a sole goal, rather improving the quality of international cooperation has been a cornerstone in MIM’s work, advocating for “genuine” cooperation and specific attention has been given to the need to de-colonize the international division of labour in research. In part, this is motivated by the deeper understanding of African societies that is needed for research to have an actual effect on the malaria burden. In part it is motivated by ethical concerns visible in the africanization concept.

A comparison could be made between Malaria R&D Alliance and the African Network for Drugs and Diagnostics Innovation (ANDI). While the former’s model for cooperation seems established as discovery to be done in the North and clinical trials in SSA, the latter’s model promotes the participation in discovering, developing and manufacturing health products and in establishing functional market mechanisms. The ANDI model comprise the insights made within Theory of Science studies that has shown that research quality and sustainability depend on extensive linkages to all the actors affected by scientific work (mainly those who produce knowledge and those who are affected by the disease and its socio-political consequences). African researchers have to be part of all the steps of product research development.

## Fifth Pan African Malaria Conference, November 2009

The secretariat raised US$ 2.2 million for the MIM Conference, which attracted a record 2,500 participants from across Africa, Europe and North America. Overall, the conference had five plenary sessions (31 keynote speakers), five controversy sessions (10 speakers), 41 scientific/parallel sessions featuring 560 oral presentations and 580 posters, 43 symposia and 75 exhibitors.

An international (AMANET, MIM, WHO/TDR, MMV, MVI, PSI, WHO/AFRO, RBM, Global Health Advocacy, Global Health Strategies) MIM Conference Media and Communications Working Group was established to cover the conference before, during and well after the event. A number of articles have appeared in the local (Kenya) and international media (TV, radio, newspapers, blogs) and on partner networks featuring stories from the MIM Conference.

Against the background of the financial world crisis, the success of the fifth MIM conference needs to be recognized. But the elimination/eradication question which has been debated one day by all the delegates is a common challenge on the current agenda that deserves all the attention and the necessary financial support for all heath sectors. It is expected that the next MIM conference will bring new investigators and more positive results in this line.

## Conclusions

The analyses of scientific knowledge production that are summed up in the africanization concept show that dense links between the research object (malaria) and the researching subject (the malaria researcher) are necessary for comprehensive and relevant scientific knowledge to emerge. Marginalizing African experiences in malaria research not only means a reproduction of colonial orders, but also that these orders continuously influence science so that reductive and superficial accounts of malaria are generated. Scientific knowledge production needs to be firmly based in the local settings where malaria is experienced and treated. This means that African authorities as well as foreign research actors need to recognize the value of nationally based scientific education and programs, so that African researchers and policy makers can take an active part in all phases of research.

The challenges facing the malaria scientific community in Africa are the following: high quality research has to be conducted in institutions based in Africa; imbalance in the distribution of external funding, and the low number of African competitors have to be managed; structures for the identification of research topics relevant to both public health authorities in malaria endemic countries and the global research agenda) have to be formulated and implemented; and, finally, African based researchers have to be encouraged to publish in scientific journals with good impact factor as a recognition of good science. These are the dilemmas of the africanization of malaria research.

## Competing interests

The authors declare that they have no competing interests.
